# Lower bounds on multiple sequence alignment using exact 3-way alignment

**DOI:** 10.1186/1471-2105-8-140

**Published:** 2007-04-30

**Authors:** Charles J Colbourn, Sudhir Kumar

**Affiliations:** 1 Center for Evolutionary Functional Genomics, The Biodesign Institute, Arizona State University, PO Box 875301, Tempe, AZ 85287-5301, USA; 2 School of Computing and Informatics, Arizona State University, PO Box 878809, Tempe, AZ 85287-8809, USA; 3 School of Life Sciences, Arizona State University, PO Box 875301, Tempe, AZ 85287-5301, USA

## Abstract

**Background:**

Multiple sequence alignment is fundamental. Exponential growth in computation time appears to be inevitable when an optimal alignment is required for many sequences. Exact costs of optimum alignments are therefore rarely computed. Consequently much effort has been invested in algorithms for alignment that are heuristic, or explore a restricted class of solutions. These give an upper bound on the alignment cost, but it is equally important to determine the quality of the solution obtained. In the absence of an optimal alignment with which to compare, lower bounds may be calculated to assess the quality of the alignment. As more effort is invested in improving upper bounds (alignment algorithms), it is therefore important to improve lower bounds as well. Although numerous cost metrics can be used to determine the quality of an alignment, many are based on sum-of-pairs (SP) measures and their generalizations.

**Results:**

Two standard and two new methods are considered for using exact 2-way and 3-way alignments to compute lower bounds on total SP alignment cost; one new method fares well with respect to accuracy, while the other reduces the computation time. The first employs exhaustive computation of exact 3-way alignments, while the second employs an efficient heuristic to compute a much smaller number of exact 3-way alignments. Calculating all 3-way alignments exactly and computing their average improves lower bounds on sum of SP cost in *v*-way alignments. However judicious selection of a subset of all 3-way alignments can yield a further improvement with minimal additional effort. On the other hand, a simple heuristic to select a random subset of 3-way alignments (a random packing) yields accuracy comparable to averaging all 3-way alignments with substantially less computational effort.

**Conclusion:**

Calculation of lower bounds on SP cost (and thus the quality of an alignment) can be improved by employing a mixture of 3-way and 2-way alignments.

## Background

Let Σ be a finite alphabet, and consider *v *words (sequences) *w*_1_,..., *w_v _*over Σ. For 1 ≤ *i *≤ *v*, let ℓ_*i *_denote the length of word *w*_*i*_, and let *σ*_*ij *_denote the *j*th character of *w*_*i*_. A string *x*_*i *_over Σ ∪ {-} is an *extension *of *w*_*i *_of length *M *if there exist indices 1 ≤ *d*_1 _<*d*_2 _< ⋯ dℓi
 MathType@MTEF@5@5@+=feaafiart1ev1aaatCvAUfKttLearuWrP9MDH5MBPbIqV92AaeXatLxBI9gBaebbnrfifHhDYfgasaacH8akY=wiFfYdH8Gipec8Eeeu0xXdbba9frFj0=OqFfea0dXdd9vqai=hGuQ8kuc9pgc9s8qqaq=dirpe0xb9q8qiLsFr0=vr0=vr0dc8meaabaqaciaacaGaaeqabaqabeGadaaakeaacqWGKbazdaWgaaWcbaGaeS4eHW2aaSbaaWqaaiabdMgaPbqabaaaleqaaaaa@30ED@ ≤ *M *so that, for 1 ≤ *j *≤ ℓ_*i*_, the *d*_*j*_th character of *x*_*i *_is *σ*_*ij*_, and whenever *j *∉ {*d*_1_,..., dℓi
 MathType@MTEF@5@5@+=feaafiart1ev1aaatCvAUfKttLearuWrP9MDH5MBPbIqV92AaeXatLxBI9gBaebbnrfifHhDYfgasaacH8akY=wiFfYdH8Gipec8Eeeu0xXdbba9frFj0=OqFfea0dXdd9vqai=hGuQ8kuc9pgc9s8qqaq=dirpe0xb9q8qiLsFr0=vr0=vr0dc8meaabaqaciaacaGaaeqabaqabeGadaaakeaacqWGKbazdaWgaaWcbaGaeS4eHW2aaSbaaWqaaiabdMgaPbqabaaaleqaaaaa@30ED@}, the *j*th character of *x*_*i *_is -. Let *Ext*(*w*_*i*_, *M*) be the set of all extensions of word *w*_*i *_to length *M*.

An *alignment *of two words *w*_*i *_and *w*_*j *_consists of an extension *x*_*i *_∈ *Ext*(*w*_*i*_, *M*) and an extension *x*_*j *_∈ *Ext*(*w*_*j*_, *M*). The *cost *of such an alignment, denoted *cost*(*x*_*i*_, *x*_*j*_), is determined position by position. In the simplest case, a position contributes a cost of 1 if the extensions do not have the same character in that position, 0 otherwise. Numerous different cost measures are of interest, but for our purposes counting matches of a symbol of Σ against – (*indels*) and matches of two different symbols of Σ (*substitutions*) suffices.

*Pairwise alignment *of sequences *w*_*i *_and *w*_*j *_chooses *M*, *x*_*i *_∈ *Ext*(*w*_*i*_, *M*) and *x*_*j *_∈ *Ext*(*w*_*j*_, *M*), to obtain an alignment with SP cost *cost*(*x*_*i*_, *x*_*j*_). The alignment with lowest cost has SP cost

min_*M*≥0 _min{*cost*(*x*_*i*_, *x*_*j*_) s.t. *x*_*i *_∈ *Ext*(*w*_*i*_, *M*), *x*_*j *_∈ *Ext*(*w*_*j*_, *M*)}.

The alignment of *v *sequences *w*_1_,..., *w*_*v *_consists of extensions {*x*_*i *_∈ *Ext*(*w*_*i*_, *M*) : 1 ≤ *i *≤ *v*}. The *sum-of-pairs *or *SP *cost is ∑_1≤*i*<*j*≤*v *_*cost*(*x*_*i*_, *x*_*j*_), and the multiple alignment with lowest cost has cost

min⁡M≥0min⁡{∑1≤i<j≤vcost(xi,xj) s.t. xi∈Ext(wi,M)∀1≤i≤v}.
 MathType@MTEF@5@5@+=feaafiart1ev1aaatCvAUfKttLearuWrP9MDH5MBPbIqV92AaeXatLxBI9gBaebbnrfifHhDYfgasaacH8akY=wiFfYdH8Gipec8Eeeu0xXdbba9frFj0=OqFfea0dXdd9vqai=hGuQ8kuc9pgc9s8qqaq=dirpe0xb9q8qiLsFr0=vr0=vr0dc8meaabaqaciaacaGaaeqabaqabeGadaaakeaacyGGTbqBcqGGPbqAcqGGUbGBdaWgaaWcbaGaemyta0KaeyyzImRaeGimaadabeaakiGbc2gaTjabcMgaPjabc6gaUjabcUha7naaqafabaacbiGae83yamMae83Ba8Mae83CamNaemiDaqNaeiikaGIaemiEaG3aaSbaaSqaaiabdMgaPbqabaGccqGGSaalcqWG4baEdaWgaaWcbaGaemOAaOgabeaakiabcMcaPiabbccaGiabbohaZjabb6caUiabbsha0jabb6caUiabbccaGiabdIha4naaBaaaleaacqWGPbqAaeqaaOGaeyicI4SaemyrauKaemiEaGNaemiDaqNaeiikaGIaem4DaC3aaSbaaSqaaiabdMgaPbqabaGccqGGSaalcqWGnbqtcqGGPaqkcqGHaiIicqaIXaqmcqGHKjYOcqWGPbqAcqGHKjYOcqWG2bGDcqGG9bqFcqGGUaGlaSqaaiabigdaXiabgsMiJkabdMgaPjabgYda8iabdQgaQjabgsMiJkabdAha2bqab0GaeyyeIuoaaaa@743E@

One natural objective is to find a multiple alignment that minimizes the SP cost. Exact methods have, for the most part, employed dynamic programming. In this environment, the execution time grows polynomially with the length of the sequences, but exponentially in the number of sequences. While improvements in time and space utilization are possible (for example, [1-7]), dynamic programming remains infeasible for large numbers of sequences. Indeed, finding optimal multiple sequence alignments under the SP cost metric is NP-hard [8-10]. Consequently, substantial effort has been invested in finding effective heuristic algorithms (see [4,11-15] for surveys). These heuristic methods produce upper bounds on the cost of a multiple sequence alignment with minimum SP cost.

When heuristic methods are employed, how can one assess the quality of the alignment obtained? Feng and Doolittle [16] argue that the main biological motivation for multiple sequence alignment is to reconstruct phylogeny; see [17] for a recent overview. With this in mind, quality relates to the amount of biological information revealed by the alignment. Let us ask a more modest question. How well does the SP cost produced by a specific method compare to the minimum SP cost over all alignments? In other words, what is the accuracy of the upper bound? A precise answer poses a significant challenge; comparison with an optimal solution would require that one be found, negating the need for the comparison. It is natural, therefore, to determine a *lower *bound on the SP cost, and compare the heuristic method with the lower bound as a measure of accuracy. Lower bounds do not provide alignments; instead, when they are in good agreement with an upper bound, they certify that the upper bound is indeed close to optimum. Lower bounds are also employed in branch-and-bound strategies for exact alignment [18,19], but our focus is on tools for comparison with upper bounds. A comparison of an upper and a lower bound simultaneously measures the accuracy of the upper bound, and the lower bound. No matter how good an upper bound is, comparison with a poor lower bound fails to certify that the upper bound is close to optimum. Therefore improvements in lower bounds are crucial, not to reveal biological information through alignment directly, but rather to better calibrate an heuristic method that is being developed for biological inferences. Determining effective *lower *bounds on the SP cost of a multiple sequence alignment has been much less studied than heuristic methods, primarily because a lower bound does not provide a feasible alignment. Nevertheless, an effective lower bound provides the only real means to assess proximity of an upper bound to the optimum. In this paper, we examine a conceptually simple technique for calculating lower bounds on SP costs for alignments. It is based on the observation that *v*-way alignments for small values of *v *can be efficiently calculated by dynamic programming. The specific methods that we propose combines exact 2- and 3-way alignments selected to treat each 2-way alignment exactly once. Randomization of the selection of 3-way alignments to examine enables us to improve lower bounds from 2-way alignments without excessive computational cost.

## Results and Discussion

The minimum SP cost, *mincost*({*w*_1_,..., *w*_*v*_}), is

min⁡M≥0min⁡{∑1≤i<j≤vcost(xi,xj) s.t. xi∈Ext(wi,M)∀1≤i≤v}.
 MathType@MTEF@5@5@+=feaafiart1ev1aaatCvAUfKttLearuWrP9MDH5MBPbIqV92AaeXatLxBI9gBaebbnrfifHhDYfgasaacH8akY=wiFfYdH8Gipec8Eeeu0xXdbba9frFj0=OqFfea0dXdd9vqai=hGuQ8kuc9pgc9s8qqaq=dirpe0xb9q8qiLsFr0=vr0=vr0dc8meaabaqaciaacaGaaeqabaqabeGadaaakeaacyGGTbqBcqGGPbqAcqGGUbGBdaWgaaWcbaGaemyta0KaeyyzImRaeGimaadabeaakiGbc2gaTjabcMgaPjabc6gaUjabcUha7naaqafabaacbiGae83yamMae83Ba8Mae83CamNaemiDaqNaeiikaGIaemiEaG3aaSbaaSqaaiabdMgaPbqabaGccqGGSaalcqWG4baEdaWgaaWcbaGaemOAaOgabeaakiabcMcaPiabbccaGiabbohaZjabb6caUiabbsha0jabb6caUiabbccaGiabdIha4naaBaaaleaacqWGPbqAaeqaaOGaeyicI4SaemyrauKaemiEaGNaemiDaqNaeiikaGIaem4DaC3aaSbaaSqaaiabdMgaPbqabaGccqGGSaalcqWGnbqtcqGGPaqkcqGHaiIicqaIXaqmcqGHKjYOcqWGPbqAcqGHKjYOcqWG2bGDcqGG9bqFcqGGUaGlaSqaaiabigdaXiabgsMiJkabdMgaPjabgYda8iabdQgaQjabgsMiJkabdAha2bqab0GaeyyeIuoaaaa@743E@

A simple lower bound is obtained by treating the alignment as (v2)
 MathType@MTEF@5@5@+=feaafiart1ev1aaatCvAUfKttLearuWrP9MDH5MBPbIqV92AaeXatLxBI9gBaebbnrfifHhDYfgasaacH8akY=wiFfYdH8Gipec8Eeeu0xXdbba9frFj0=OqFfea0dXdd9vqai=hGuQ8kuc9pgc9s8qqaq=dirpe0xb9q8qiLsFr0=vr0=vr0dc8meaabaqaciaacaGaaeqabaqabeGadaaakeaadaqadaqaauaabeqaceaaaeaacqWG2bGDaeaacqaIYaGmaaaacaGLOaGaayzkaaaaaa@30A9@ independent 2-way alignments [3]:

mincost({w1,...,wv})≥∑1≤i<j≤vmin⁡M≥0{cost(xi,xj) s.t. xi,xj∈Ext(wi,M)}
 MathType@MTEF@5@5@+=feaafiart1ev1aaatCvAUfKttLearuWrP9MDH5MBPbIqV92AaeXatLxBI9gBaebbnrfifHhDYfgasaacH8akY=wiFfYdH8Gipec8Eeeu0xXdbba9frFj0=OqFfea0dXdd9vqai=hGuQ8kuc9pgc9s8qqaq=dirpe0xb9q8qiLsFr0=vr0=vr0dc8meaabaqaciaacaGaaeqabaqabeGadaaakeaaieGacqWFTbqBcqWFPbqAcqWFUbGBcqWFJbWycqWFVbWBcqWFZbWCcqWG0baDcqGGOaakcqGG7bWEcqWG3bWDdaWgaaWcbaGaeGymaedabeaakiabcYcaSiabc6caUiabc6caUiabc6caUiabcYcaSiabdEha3naaBaaaleaacqWG2bGDaeqaaOGaeiyFa0NaeiykaKIaeyyzIm7aaabuaeaacyGGTbqBcqGGPbqAcqGGUbGBdaWgaaWcbaGaemyta0KaeyyzImRaeGimaadabeaakiabcUha7jab=ngaJjab=9gaVjab=nhaZjabdsha0jabcIcaOiabdIha4naaBaaaleaacqWGPbqAaeqaaOGaeiilaWIaemiEaG3aaSbaaSqaaiabdQgaQbqabaGccqGGPaqkcqqGGaaicqqGZbWCcqqGUaGlcqqG0baDcqqGUaGlcqqGGaaicqWG4baEdaWgaaWcbaGaemyAaKgabeaakiabcYcaSiabdIha4naaBaaaleaacqWGQbGAaeqaaOGaeyicI4SaemyrauKaemiEaGNaemiDaqNaeiikaGIaem4DaC3aaSbaaSqaaiabdMgaPbqabaGccqGGSaalcqWGnbqtcqGGPaqkcqGG9bqFaSqaaiabigdaXiabgsMiJkabdMgaPjabgYda8iabdQgaQjabgsMiJkabdAha2bqab0GaeyyeIuoaaaa@856E@

By way of example, consider the eighteen sequences given next; these are generated from a sequence of 50 bases by random insertions, deletions, and substitutions. Our interest is in determining a lower bound on the sum of SP costs in an 18-way alignment of these sequences (see Table 1).

**Table 1:** 

Sequence #	Sequence
1	ATGGGTTGCGTCAGGAGTAAAGAAGCCAAGGGCCCGGCACTGAAGTACCA
2	ATGGGCTGTATTAAAAGTAAGGAAGACAAAGGACCAGCAATCAAGTACAG
3	ATGGGCTGCATTAAAAGTAAACAAAAGTCCAGCCATAAAATACAC
4	ATGGGCTGCATTAAAAGTAAAGAAAACAAAAGTCCAGCCATTAAATACAG
5	AGGGGTGCATTAAAAGCAAAGAAGATAAAGGTCCAGCCATGAAATACAG
6	ATGCTGTATAAAAAGTAAGGAAGACAAAGGACCATCCATCAAGTACAG
7	ATGAGTTGCGTCAGGAGTAGAGAGCTAAGGGCCCGGCACTGAAGTACCA
8	ATGGGTTGCATTAGAAGTAAAGAAAACAGAAGTCCAGCCATCAAATACAC
9	ATGAGCTGTATTAAGTAAGGAAGACAAAGGACCAGCAATGAAGTACAG
10	ATGAGCTGCATTAAAAGTAAAGAAAACAAAAGTCCAGCCATTAAATACAG
11	ATGAGGTGCATTAATAGCAAAGAAGATACAGGTCCAGCCATGATACAG
12	AGAGCTGTATAAAGAGTAAGGAGACAAAGGACATCCATCAAGTACAA
13	ATGGCTTGCGTCAGGAGTAAAGAAGCCAAGGGCCCAGCACTGAAGTACCA
14	ATGGGCTGCATTAAAGTAAAGACAAAAGTCCAGCCATGAAATACAC
15	ATGGGCTGTATTAAGAGTAAGAAGACAAAGGACCAGCAATCAAGTACAG
16	AGTGGCTGCATTAAAAGTAATGAAAACAAAAGTCCAGCCATTAAATACAT
17	ATGGTGTGCATTAGAAGTAAAGAAGACAGAAGTCCAGCCATCATATAGAC
18	AGACGTGTATACAGAGTAAGGAGACAAAGGACATCCATCAAGTACAG

Using dynamic programming on the 153 = (182)
 MathType@MTEF@5@5@+=feaafiart1ev1aaatCvAUfKttLearuWrP9MDH5MBPbIqV92AaeXatLxBI9gBaebbnrfifHhDYfgasaacH8akY=wiFfYdH8Gipec8Eeeu0xXdbba9frFj0=OqFfea0dXdd9vqai=hGuQ8kuc9pgc9s8qqaq=dirpe0xb9q8qiLsFr0=vr0=vr0dc8meaabaqaciaacaGaaeqabaqabeGadaaakeaadaqadaqaauaabeqaceaaaeaacqaIXaqmcqaI4aaoaeaacqaIYaGmaaaacaGLOaGaayzkaaaaaa@3122@ pairs, the sum of 2-way alignments gives a total cost of 1946.

### Fractional Alignment

This decomposition of the (v2)
 MathType@MTEF@5@5@+=feaafiart1ev1aaatCvAUfKttLearuWrP9MDH5MBPbIqV92AaeXatLxBI9gBaebbnrfifHhDYfgasaacH8akY=wiFfYdH8Gipec8Eeeu0xXdbba9frFj0=OqFfea0dXdd9vqai=hGuQ8kuc9pgc9s8qqaq=dirpe0xb9q8qiLsFr0=vr0=vr0dc8meaabaqaciaacaGaaeqabaqabeGadaaakeaadaqadaqaauaabeqaceaaaeaacqWG2bGDaeaacqaIYaGmaaaacaGLOaGaayzkaaaaaa@30A9@ simultaneous pairwise alignments into (v2)
 MathType@MTEF@5@5@+=feaafiart1ev1aaatCvAUfKttLearuWrP9MDH5MBPbIqV92AaeXatLxBI9gBaebbnrfifHhDYfgasaacH8akY=wiFfYdH8Gipec8Eeeu0xXdbba9frFj0=OqFfea0dXdd9vqai=hGuQ8kuc9pgc9s8qqaq=dirpe0xb9q8qiLsFr0=vr0=vr0dc8meaabaqaciaacaGaaeqabaqabeGadaaakeaadaqadaqaauaabeqaceaaaeaacqWG2bGDaeaacqaIYaGmaaaacaGLOaGaayzkaaaaaa@30A9@ independent 2-way alignment problems can be generalized. To do so, select a number *α *of classes in the decomposition. Then for 1 ≤ *c *≤ *α *and pair {*i*, *j*} with 1 ≤ *i *<*j *≤ *v*, define a *weight μ*_*c*, {*i*, *j*}_. It is a *decomposition *when, for every class *c*, ∑c=1αμc,{i,j}=1
 MathType@MTEF@5@5@+=feaafiart1ev1aaatCvAUfKttLearuWrP9MDH5MBPbIqV92AaeXatLxBI9gBaebbnrfifHhDYfgasaacH8akY=wiFfYdH8Gipec8Eeeu0xXdbba9frFj0=OqFfea0dXdd9vqai=hGuQ8kuc9pgc9s8qqaq=dirpe0xb9q8qiLsFr0=vr0=vr0dc8meaabaqaciaacaGaaeqabaqabeGadaaakeaadaaeWaqaaGGaciab=X7aTnaaBaaaleaacqWGJbWycqGGSaalcqGG7bWEcqWGPbqAcqGGSaalcqWGQbGAcqGG9bqFaeqaaaqaaiabdogaJjabg2da9iabigdaXaqaaiab=f7aHbqdcqGHris5aOGaeyypa0JaeGymaedaaa@4032@ for every pair {*i*, *j*}. For a given class *c *in such a decomposition,

min⁡M≥0min⁡{∑1≤i<j≤vμc,{i,j}cost(xi,xj) s.t. xi∈Ext(wi,M)∀1≤i≤v}
 MathType@MTEF@5@5@+=feaafiart1ev1aaatCvAUfKttLearuWrP9MDH5MBPbIqV92AaeXatLxBI9gBaebbnrfifHhDYfgasaacH8akY=wiFfYdH8Gipec8Eeeu0xXdbba9frFj0=OqFfea0dXdd9vqai=hGuQ8kuc9pgc9s8qqaq=dirpe0xb9q8qiLsFr0=vr0=vr0dc8meaabaqaciaacaGaaeqabaqabeGadaaakeaacyGGTbqBcqGGPbqAcqGGUbGBdaWgaaWcbaGaemyta0KaeyyzImRaeGimaadabeaakiGbc2gaTjabcMgaPjabc6gaUnaacmqabaWaaabuaeaaiiGacqWF8oqBdaWgaaWcbaGaem4yamMaeiilaWIaei4EaSNaemyAaKMaeiilaWIaemOAaOMaeiyFa0habeaaieGakiab+ngaJjab+9gaVjab+nhaZjabdsha0jabcIcaOiabdIha4naaBaaaleaacqWGPbqAaeqaaOGaeiilaWIaemiEaG3aaSbaaSqaaiabdQgaQbqabaGccqGGPaqkcqqGGaaicqqGZbWCcqqGUaGlcqqG0baDcqqGUaGlcqqGGaaicqWG4baEdaWgaaWcbaGaemyAaKgabeaakiabgIGiolabdweafjabdIha4jabdsha0jabcIcaOiabdEha3naaBaaaleaacqWGPbqAaeqaaOGaeiilaWIaemyta0KaeiykaKIaeyiaIiIaeGymaeJaeyizImQaemyAaKMaeyizImQaemODayhaleaacqaIXaqmcqGHKjYOcqWGPbqAcqGH8aapcqWGQbGAcqGHKjYOcqWG2bGDaeqaniabggHiLdaakiaawUhacaGL9baaaaa@7D4D@

gives a *fractional *SP alignment cost, with the contribution of each pair scaled by its weight in the class of the decomposition. Indeed

∑c=1αmin⁡M≥0min⁡{∑1≤i<j≤vμc,{i,j}cost(xi,xj) s.t. xi∈Ext(wi,M)∀1≤i≤v}
 MathType@MTEF@5@5@+=feaafiart1ev1aaatCvAUfKttLearuWrP9MDH5MBPbIqV92AaeXatLxBI9gBaebbnrfifHhDYfgasaacH8akY=wiFfYdH8Gipec8Eeeu0xXdbba9frFj0=OqFfea0dXdd9vqai=hGuQ8kuc9pgc9s8qqaq=dirpe0xb9q8qiLsFr0=vr0=vr0dc8meaabaqaciaacaGaaeqabaqabeGadaaakeaadaaeWbqaaiGbc2gaTjabcMgaPjabc6gaUnaaBaaaleaacqWGnbqtcqGHLjYScqaIWaamaeqaaOGagiyBa0MaeiyAaKMaeiOBa4galeaacqWGJbWycqGH9aqpcqaIXaqmaeaaiiGacqWFXoqya0GaeyyeIuoakmaacmqabaWaaabuaeaacqWF8oqBdaWgaaWcbaGaem4yamMaeiilaWIaei4EaSNaemyAaKMaeiilaWIaemOAaOMaeiyFa0habeaaieGakiab+ngaJjab+9gaVjab+nhaZjabdsha0jabcIcaOiabdIha4naaBaaaleaacqWGPbqAaeqaaOGaeiilaWIaemiEaG3aaSbaaSqaaiabdQgaQbqabaGccqGGPaqkcqqGGaaicqqGZbWCcqqGUaGlcqqG0baDcqqGUaGlcqqGGaaicqWG4baEdaWgaaWcbaGaemyAaKgabeaakiabgIGiolabdweafjabdIha4jabdsha0jabcIcaOiabdEha3naaBaaaleaacqWGPbqAaeqaaOGaeiilaWIaemyta0KaeiykaKIaeyiaIiIaeGymaeJaeyizImQaemyAaKMaeyizImQaemODayhaleaacqaIXaqmcqGHKjYOcqWGPbqAcqGH8aapcqWGQbGAcqGHKjYOcqWG2bGDaeqaniabggHiLdaakiaawUhacaGL9baaaaa@8478@

is a lower bound on the minimum SP *v*-way alignment cost for any decomposition.

That this is a lower bound on *mincost*({*w*_1_,..., *w*_*v*_}) is easy to demonstrate. When the decomposition has a single class, the lower bound given is equal to the SP alignment cost. So consider two classes in the decomposition *c' *and *c"*. Merge these two classes to form a single class *c *by setting *μ*_*c*, {*i*, *j*} _= *μ*_*c'*, {*i*, *j*} _+ *μ*_*c"*, {*i*, *j*} _for every pair {*i*, *j*}, removing classes *c' *and *c"*. We need only verify that, when *M *is chosen to yield the best fractional alignment for class *c *(i.e. the extensions *x*_1_,..., *x*_*v*_),

min⁡xi,yi,zi∈Ext(wi,M)∀1≤i≤v[∑1≤i<j≤vμc′{i,j}(cost(xi,xj)−cost(yi,yj))+μc″,{i,j}(cost(xi,xj)−cost(zi,zj))]≥0
 MathType@MTEF@5@5@+=feaafiart1ev1aaatCvAUfKttLearuWrP9MDH5MBPbIqV92AaeXatLxBI9gBaebbnrfifHhDYfgasaacH8akY=wiFfYdH8Gipec8Eeeu0xXdbba9frFj0=OqFfea0dXdd9vqai=hGuQ8kuc9pgc9s8qqaq=dirpe0xb9q8qiLsFr0=vr0=vr0dc8meaabaqaciaacaGaaeqabaqabeGadaaakeaacyGGTbqBcqGGPbqAcqGGUbGBdaWgaaWcbaGaemiEaG3aaSbaaWqaaiabdMgaPbqabaWccqGGSaalcqWG5bqEdaWgaaadbaGaemyAaKgabeaaliabcYcaSiabdQha6naaBaaameaacqWGPbqAaeqaaSGaeyicI4SaemyrauKaemiEaGNaemiDaqNaeiikaGIaem4DaC3aaSbaaWqaaiabdMgaPbqabaWccqGGSaalcqWGnbqtcqGGPaqkcqGHaiIicqaIXaqmcqGHKjYOcqWGPbqAcqGHKjYOcqWG2bGDaeqaaOWaamWaaeaadaaeqbqaaGGaciab=X7aTnaaBaaaleaacuWGJbWygaqbaiabcUha7jabdMgaPjabcYcaSiabdQgaQjabc2ha9bqabaGccqGGOaakieGacqGFJbWycqGFVbWBcqGFZbWCcqWG0baDcqGGOaakcqWG4baEdaWgaaWcbaGaemyAaKgabeaakiabcYcaSiabdIha4naaBaaaleaacqWGQbGAaeqaaOGaeiykaKIaeyOeI0Iae43yamMae43Ba8Mae43CamNaemiDaqNaeiikaGIaemyEaK3aaSbaaSqaaiabdMgaPbqabaGccqGGSaalcqWG5bqEdaWgaaWcbaGaemOAaOgabeaakiabcMcaPiabcMcaPiabgUcaRiab=X7aTnaaBaaaleaacuWGJbWygaGbaiabcYcaSiabcUha7jabdMgaPjabcYcaSiabdQgaQjabc2ha9bqabaGccqGGOaakcqGFJbWycqGFVbWBcqGFZbWCcqWG0baDcqGGOaakcqWG4baEdaWgaaWcbaGaemyAaKgabeaakiabcYcaSiabdIha4naaBaaaleaacqWGQbGAaeqaaOGaeiykaKIaeyOeI0Iae43yamMae43Ba8Mae43CamNaemiDaqNaeiikaGIaemOEaO3aaSbaaSqaaiabdMgaPbqabaGccqGGSaalcqWG6bGEdaWgaaWcbaGaemOAaOgabeaakiabcMcaPiabcMcaPaWcbaGaeGymaeJaeyizImQaemyAaKMaeyipaWJaemOAaOMaeyizImQaemODayhabeqdcqGHris5aaGccaGLBbGaayzxaaGaeyyzImRaeGimaadaaa@B41A@

where *y*_1_,..., *y*_*v *_form a best fractional alignment (of length M) for *c' *and *z*_1_,..., *z*_*v *_a best for *c"*. In applying this general framework, one must calculate fractional alignment costs, and this appears to necessitate solving many *v*-way alignment problems. Each can be solved by dynamic programming, but unless the decomposition is suitably chosen the resulting subproblems may be no simpler to solve than the original. If, in a given class of the decomposition, most weights are zero, the fractional alignment problem is substantially simplified. In particular, if all pairs containing a particular sequence have weights equal to zero in this class, the sequence does not participate in the fractional alignment. Then by choosing weights within each class so that "few" sequences appear in pairs with nonzero weight, the alignment task is reduced to one on the few sequences only, and can be solved efficiently using dynamic programming. Indeed when *α *= (v2)
 MathType@MTEF@5@5@+=feaafiart1ev1aaatCvAUfKttLearuWrP9MDH5MBPbIqV92AaeXatLxBI9gBaebbnrfifHhDYfgasaacH8akY=wiFfYdH8Gipec8Eeeu0xXdbba9frFj0=OqFfea0dXdd9vqai=hGuQ8kuc9pgc9s8qqaq=dirpe0xb9q8qiLsFr0=vr0=vr0dc8meaabaqaciaacaGaaeqabaqabeGadaaakeaadaqadaqaauaabeqaceaaaeaacqWG2bGDaeaacqaIYaGmaaaacaGLOaGaayzkaaaaaa@30A9@ and every class *c *has *μ*_*c*, {*i*, *j*} _= 1 for exactly one pair {*i*, *j*}, this is the decomposition of the *v*-way alignment into (v2)
 MathType@MTEF@5@5@+=feaafiart1ev1aaatCvAUfKttLearuWrP9MDH5MBPbIqV92AaeXatLxBI9gBaebbnrfifHhDYfgasaacH8akY=wiFfYdH8Gipec8Eeeu0xXdbba9frFj0=OqFfea0dXdd9vqai=hGuQ8kuc9pgc9s8qqaq=dirpe0xb9q8qiLsFr0=vr0=vr0dc8meaabaqaciaacaGaaeqabaqabeGadaaakeaadaqadaqaauaabeqaceaaaeaacqWG2bGDaeaacqaIYaGmaaaacaGLOaGaayzkaaaaaa@30A9@ independent 2-way alignments discussed earlier. The decomposition framework suggests a means to improve this simple bound, which we pursue next.

### Improvement by 3-way alignment

There is every reason to expect that the best alignment of two sequences may conflict with the best 2-way alignments of each with a third. For example, aligning the first three sequences of the eighteen in the example optimally, one obtains the alignment

ATGGGTTGCGTCAGGAGTAAAGAAGCCAAGGGCCCGGCACTGAAGTACCA

ATGGGCTGTATTAAAAGTAAGGAAGACAAAGGACCAGCAATCAAGTACAG

ATGGGCTGCATTAAAAGTAAACAA---AA-GT-CCAGCCATAAAATACAC

Between the first and second sequences, there are 15 substitutions and no indels. Between the second and third, there are 8 substitutions and 5 indels. Between the first and third there are 14 substitutions and 5 indels. The total three-way alignment score is 15 + 13 + 19 = 47. When exact two-way alignment of each pair among the three sequences is done, the alignment of the first two sequences agrees with that in the 3-way alignment. The optimal alignment of the second and third sequences does not; it has cost 12 (shown with 7 substitutions and 5 indels):

ATGGGCTGTATTAAAAGTAAGGAAGACAAAGGACCAGCAATCAAGTACAG

ATGGGCTGCATTAAAAGT----AA-ACAAAAGTCCAGCCATAAAATACAC

Moreover, the optimal 2-way alignment for the first and third sequences has cost 18 (shown as 13 substitutions and 5 indels):

ATGGGTTGCGTCAGGAGTAAAGAAGCCAAGGGCCCGGCACTGAAGTACCA

ATGGGCTGCATTAAAAGTAAACAA---AA-GTCCAGCCA-TAAAATACAC

The sum of the three optimal 2-way alignment costs is therefore 45, while the optimal 3-way alignment has cost 47. Hence the use of exact 3-way alignment provides a better bound; thus rather than computing the lower bound by adding the SP scores for the three pairs of sequences, one can employ an exact 3-way alignment for all three sequences. As shown in the example, the difference in SP scores of simultaneous three-way alignment of the three sequences and sum of pairwise scores of the three pairs in this triple is 2. In this example, the best improvement that can be achieved by replacing any three 2-way alignments by the corresponding 3-way alignment is 3. To establish this, all 816 3-way alignments were calculated using dynamic programming, and compared to the sum of 2-way alignments within each.

### Average 3-way alignment

In *v*-way alignment, a reasonable goal therefore is to choose many triples of sequences for which the 3-way alignment improves the lower bound, so as to obtain many small improvements that aggregate to form a large improvement in the bound. At the extreme, one could calculate best 3-way alignments for all (v3)
 MathType@MTEF@5@5@+=feaafiart1ev1aaatCvAUfKttLearuWrP9MDH5MBPbIqV92AaeXatLxBI9gBaebbnrfifHhDYfgasaacH8akY=wiFfYdH8Gipec8Eeeu0xXdbba9frFj0=OqFfea0dXdd9vqai=hGuQ8kuc9pgc9s8qqaq=dirpe0xb9q8qiLsFr0=vr0=vr0dc8meaabaqaciaacaGaaeqabaqabeGadaaakeaadaqadaqaauaabeqaceaaaeaacqWG2bGDaeaacqaIZaWmaaaacaGLOaGaayzkaaaaaa@30AB@ triples of sequences. This corresponds to a decomposition of the *v*-way alignment into (v3)
 MathType@MTEF@5@5@+=feaafiart1ev1aaatCvAUfKttLearuWrP9MDH5MBPbIqV92AaeXatLxBI9gBaebbnrfifHhDYfgasaacH8akY=wiFfYdH8Gipec8Eeeu0xXdbba9frFj0=OqFfea0dXdd9vqai=hGuQ8kuc9pgc9s8qqaq=dirpe0xb9q8qiLsFr0=vr0=vr0dc8meaabaqaciaacaGaaeqabaqabeGadaaakeaadaqadaqaauaabeqaceaaaeaacqWG2bGDaeaacqaIZaWmaaaacaGLOaGaayzkaaaaaa@30AB@ classes; in each the three pairs of a triple are given weight 1v−2
 MathType@MTEF@5@5@+=feaafiart1ev1aaatCvAUfKttLearuWrP9MDH5MBPbIqV92AaeXatLxBI9gBaebbnrfifHhDYfgasaacH8akY=wiFfYdH8Gipec8Eeeu0xXdbba9frFj0=OqFfea0dXdd9vqai=hGuQ8kuc9pgc9s8qqaq=dirpe0xb9q8qiLsFr0=vr0=vr0dc8meaabaqaciaacaGaaeqabaqabeGadaaakeaadaWcaaqaaiabigdaXaqaaiabdAha2jabgkHiTiabikdaYaaaaaa@3100@ and the remaining weights are 0. In the example, the SP lower bound using all 2-way alignments is 1946; using all 816 3-way alignments improves the lower bound to 1969. Of the 816 3-way alignments, 510 yield no improvement; 251 yield an improvement of 1; 48 an improvement of 2; and 7 an improvement of 3. The average improvement is therefore (251·1 + 48·2 + 7·3)/16 = 23.

There are two problems with this approach. First and foremost, while some triples yield improved cost bounds over the sum of the three pairs of the triple, others may not. Simply averaging the contributions of each negates to a degree the improvements attributable to some triples. Secondly, in addition to a substantial increase in cost for each alignment, the calculation involves completing *O*(*v*^3^) alignments rather than *O*(*v*^2^). It is therefore desirable to reduce the number of classes in the decomposition, both to reduce computation time and to potentially improve the bound.

### Weighted selection of 3-way alignments

We now employ only some of the 3-way alignments. Choose a collection T
 MathType@MTEF@5@5@+=feaafiart1ev1aaatCvAUfKttLearuWrP9MDH5MBPbIqV92AaeXatLxBI9gBamrtHrhAL1wy0L2yHvtyaeHbnfgDOvwBHrxAJfwnaebbnrfifHhDYfgasaacH8akY=wiFfYdH8Gipec8Eeeu0xXdbba9frFj0=OqFfea0dXdd9vqai=hGuQ8kuc9pgc9s8qqaq=dirpe0xb9q8qiLsFr0=vr0=vr0dc8meaabaqaciaacaGaaeqabaWaaeGaeaaakeaaimaacqWFtepvaaa@3847@ of triples. For a pair {*i*, *j*} of sequences, let *λ*_*ij *_be the number of triples of T
 MathType@MTEF@5@5@+=feaafiart1ev1aaatCvAUfKttLearuWrP9MDH5MBPbIqV92AaeXatLxBI9gBamrtHrhAL1wy0L2yHvtyaeHbnfgDOvwBHrxAJfwnaebbnrfifHhDYfgasaacH8akY=wiFfYdH8Gipec8Eeeu0xXdbba9frFj0=OqFfea0dXdd9vqai=hGuQ8kuc9pgc9s8qqaq=dirpe0xb9q8qiLsFr0=vr0=vr0dc8meaabaqaciaacaGaaeqabaWaaeGaeaaakeaaimaacqWFtepvaaa@3847@ in which it appears. We form a decomposition as follows. For each *T *∈ T
 MathType@MTEF@5@5@+=feaafiart1ev1aaatCvAUfKttLearuWrP9MDH5MBPbIqV92AaeXatLxBI9gBamrtHrhAL1wy0L2yHvtyaeHbnfgDOvwBHrxAJfwnaebbnrfifHhDYfgasaacH8akY=wiFfYdH8Gipec8Eeeu0xXdbba9frFj0=OqFfea0dXdd9vqai=hGuQ8kuc9pgc9s8qqaq=dirpe0xb9q8qiLsFr0=vr0=vr0dc8meaabaqaciaacaGaaeqabaWaaeGaeaaakeaaimaacqWFtepvaaa@3847@ we form a class c, setting *μ*_*c*, {*i*, *j*} _= 1λij
 MathType@MTEF@5@5@+=feaafiart1ev1aaatCvAUfKttLearuWrP9MDH5MBPbIqV92AaeXatLxBI9gBaebbnrfifHhDYfgasaacH8akY=wiFfYdH8Gipec8Eeeu0xXdbba9frFj0=OqFfea0dXdd9vqai=hGuQ8kuc9pgc9s8qqaq=dirpe0xb9q8qiLsFr0=vr0=vr0dc8meaabaqaciaacaGaaeqabaqabeGadaaakeaadaWcaaqaaiabigdaXaqaaGGaciab=T7aSnaaBaaaleaacqWGPbqAcqWGQbGAaeqaaaaaaaa@324B@ when {*i*, *j*} ⊂ *T*, 0 otherwise. Further, whenever there is a pair {*i*, *j*} with *λ*_*ij *_= 0, form a class in which *μ*_*c*, {*i*, *j*} _= 1 and all other weights are zero. This decomposition requires the determination of fractional 3-way alignments for each triple of T
 MathType@MTEF@5@5@+=feaafiart1ev1aaatCvAUfKttLearuWrP9MDH5MBPbIqV92AaeXatLxBI9gBamrtHrhAL1wy0L2yHvtyaeHbnfgDOvwBHrxAJfwnaebbnrfifHhDYfgasaacH8akY=wiFfYdH8Gipec8Eeeu0xXdbba9frFj0=OqFfea0dXdd9vqai=hGuQ8kuc9pgc9s8qqaq=dirpe0xb9q8qiLsFr0=vr0=vr0dc8meaabaqaciaacaGaaeqabaWaaeGaeaaakeaaimaacqWFtepvaaa@3847@, and 2-way alignments for every pair appearing in any triple of T
 MathType@MTEF@5@5@+=feaafiart1ev1aaatCvAUfKttLearuWrP9MDH5MBPbIqV92AaeXatLxBI9gBamrtHrhAL1wy0L2yHvtyaeHbnfgDOvwBHrxAJfwnaebbnrfifHhDYfgasaacH8akY=wiFfYdH8Gipec8Eeeu0xXdbba9frFj0=OqFfea0dXdd9vqai=hGuQ8kuc9pgc9s8qqaq=dirpe0xb9q8qiLsFr0=vr0=vr0dc8meaabaqaciaacaGaaeqabaWaaeGaeaaakeaaimaacqWFtepvaaa@3847@.

The determination of *fractional *3-way alignments can be avoided by choosing a collection T
 MathType@MTEF@5@5@+=feaafiart1ev1aaatCvAUfKttLearuWrP9MDH5MBPbIqV92AaeXatLxBI9gBamrtHrhAL1wy0L2yHvtyaeHbnfgDOvwBHrxAJfwnaebbnrfifHhDYfgasaacH8akY=wiFfYdH8Gipec8Eeeu0xXdbba9frFj0=OqFfea0dXdd9vqai=hGuQ8kuc9pgc9s8qqaq=dirpe0xb9q8qiLsFr0=vr0=vr0dc8meaabaqaciaacaGaaeqabaWaaeGaeaaakeaaimaacqWFtepvaaa@3847@ of triples with the property that every pair belongs in either zero or *λ *of the chosen triples. Then the weights for the classes arising from triples are all 1λ
 MathType@MTEF@5@5@+=feaafiart1ev1aaatCvAUfKttLearuWrP9MDH5MBPbIqV92AaeXatLxBI9gBaebbnrfifHhDYfgasaacH8akY=wiFfYdH8Gipec8Eeeu0xXdbba9frFj0=OqFfea0dXdd9vqai=hGuQ8kuc9pgc9s8qqaq=dirpe0xb9q8qiLsFr0=vr0=vr0dc8meaabaqaciaacaGaaeqabaqabeGadaaakeaadaWcaaqaaiabigdaXaqaaGGaciab=T7aSbaaaaa@2F67@; a 3-way alignment using weights all equal to 1 can be employed, and the result divided by *λ*, to avoid the solution of fractional alignment. Using all 2-way alignments comprises one extreme, when T
 MathType@MTEF@5@5@+=feaafiart1ev1aaatCvAUfKttLearuWrP9MDH5MBPbIqV92AaeXatLxBI9gBamrtHrhAL1wy0L2yHvtyaeHbnfgDOvwBHrxAJfwnaebbnrfifHhDYfgasaacH8akY=wiFfYdH8Gipec8Eeeu0xXdbba9frFj0=OqFfea0dXdd9vqai=hGuQ8kuc9pgc9s8qqaq=dirpe0xb9q8qiLsFr0=vr0=vr0dc8meaabaqaciaacaGaaeqabaWaaeGaeaaakeaaimaacqWFtepvaaa@3847@ = ∅. Using all 3-way alignments comprises the other extreme, when T
 MathType@MTEF@5@5@+=feaafiart1ev1aaatCvAUfKttLearuWrP9MDH5MBPbIqV92AaeXatLxBI9gBamrtHrhAL1wy0L2yHvtyaeHbnfgDOvwBHrxAJfwnaebbnrfifHhDYfgasaacH8akY=wiFfYdH8Gipec8Eeeu0xXdbba9frFj0=OqFfea0dXdd9vqai=hGuQ8kuc9pgc9s8qqaq=dirpe0xb9q8qiLsFr0=vr0=vr0dc8meaabaqaciaacaGaaeqabaWaaeGaeaaakeaaimaacqWFtepvaaa@3847@ contains all possible triples of the *v *sequences (and *λ *= *v *- 2). Other choices of T
 MathType@MTEF@5@5@+=feaafiart1ev1aaatCvAUfKttLearuWrP9MDH5MBPbIqV92AaeXatLxBI9gBamrtHrhAL1wy0L2yHvtyaeHbnfgDOvwBHrxAJfwnaebbnrfifHhDYfgasaacH8akY=wiFfYdH8Gipec8Eeeu0xXdbba9frFj0=OqFfea0dXdd9vqai=hGuQ8kuc9pgc9s8qqaq=dirpe0xb9q8qiLsFr0=vr0=vr0dc8meaabaqaciaacaGaaeqabaWaaeGaeaaakeaaimaacqWFtepvaaa@3847@ can yield larger increases in the lower bound; indeed, in our example, we can improve the bound to 1994. We pursue this next.

Let *V *be a set of elements and T
 MathType@MTEF@5@5@+=feaafiart1ev1aaatCvAUfKttLearuWrP9MDH5MBPbIqV92AaeXatLxBI9gBamrtHrhAL1wy0L2yHvtyaeHbnfgDOvwBHrxAJfwnaebbnrfifHhDYfgasaacH8akY=wiFfYdH8Gipec8Eeeu0xXdbba9frFj0=OqFfea0dXdd9vqai=hGuQ8kuc9pgc9s8qqaq=dirpe0xb9q8qiLsFr0=vr0=vr0dc8meaabaqaciaacaGaaeqabaWaaeGaeaaakeaaimaacqWFtepvaaa@3847@ a set of subsets of *V*; we associate *V *with the sequences, and every set in T
 MathType@MTEF@5@5@+=feaafiart1ev1aaatCvAUfKttLearuWrP9MDH5MBPbIqV92AaeXatLxBI9gBamrtHrhAL1wy0L2yHvtyaeHbnfgDOvwBHrxAJfwnaebbnrfifHhDYfgasaacH8akY=wiFfYdH8Gipec8Eeeu0xXdbba9frFj0=OqFfea0dXdd9vqai=hGuQ8kuc9pgc9s8qqaq=dirpe0xb9q8qiLsFr0=vr0=vr0dc8meaabaqaciaacaGaaeqabaWaaeGaeaaakeaaimaacqWFtepvaaa@3847@ with a subset of sequences to align. Then (*V*, T
 MathType@MTEF@5@5@+=feaafiart1ev1aaatCvAUfKttLearuWrP9MDH5MBPbIqV92AaeXatLxBI9gBamrtHrhAL1wy0L2yHvtyaeHbnfgDOvwBHrxAJfwnaebbnrfifHhDYfgasaacH8akY=wiFfYdH8Gipec8Eeeu0xXdbba9frFj0=OqFfea0dXdd9vqai=hGuQ8kuc9pgc9s8qqaq=dirpe0xb9q8qiLsFr0=vr0=vr0dc8meaabaqaciaacaGaaeqabaWaaeGaeaaakeaaimaacqWFtepvaaa@3847@) is a (*v*, *k*, *λ*)-*packing *when |*V*| = *v*, every set in T
 MathType@MTEF@5@5@+=feaafiart1ev1aaatCvAUfKttLearuWrP9MDH5MBPbIqV92AaeXatLxBI9gBamrtHrhAL1wy0L2yHvtyaeHbnfgDOvwBHrxAJfwnaebbnrfifHhDYfgasaacH8akY=wiFfYdH8Gipec8Eeeu0xXdbba9frFj0=OqFfea0dXdd9vqai=hGuQ8kuc9pgc9s8qqaq=dirpe0xb9q8qiLsFr0=vr0=vr0dc8meaabaqaciaacaGaaeqabaWaaeGaeaaakeaaimaacqWFtepvaaa@3847@ has size *k*, and every pair of elements in *V *appears in at most *λ *sets in T
 MathType@MTEF@5@5@+=feaafiart1ev1aaatCvAUfKttLearuWrP9MDH5MBPbIqV92AaeXatLxBI9gBamrtHrhAL1wy0L2yHvtyaeHbnfgDOvwBHrxAJfwnaebbnrfifHhDYfgasaacH8akY=wiFfYdH8Gipec8Eeeu0xXdbba9frFj0=OqFfea0dXdd9vqai=hGuQ8kuc9pgc9s8qqaq=dirpe0xb9q8qiLsFr0=vr0=vr0dc8meaabaqaciaacaGaaeqabaWaaeGaeaaakeaaimaacqWFtepvaaa@3847@. The packing is *maximal *when no set can be added to T
 MathType@MTEF@5@5@+=feaafiart1ev1aaatCvAUfKttLearuWrP9MDH5MBPbIqV92AaeXatLxBI9gBamrtHrhAL1wy0L2yHvtyaeHbnfgDOvwBHrxAJfwnaebbnrfifHhDYfgasaacH8akY=wiFfYdH8Gipec8Eeeu0xXdbba9frFj0=OqFfea0dXdd9vqai=hGuQ8kuc9pgc9s8qqaq=dirpe0xb9q8qiLsFr0=vr0=vr0dc8meaabaqaciaacaGaaeqabaWaaeGaeaaakeaaimaacqWFtepvaaa@3847@ to obtain a packing with the same parameters, and it is *maximum *when it has the most sets of any packing with these parameters. See [20,21] for results on packings

Packings with *λ *= 1, i.e. (*v*, 3, 1)-packings, trivially have the property that every pair appears either in zero or in *λ *= 1 triples of the packing, and hence an SP lower bound can be calculated directly. Since every triple in the packing contains three pairs and every pair is in at most one triple, no (*v*, 3, 1)-packing can contain more than *v*(*v *- 1)/6 triples. Using such a packing for *v*-way alignment, we therefore employ only *O*(*v*^2^) 3-way alignments, rather than the *O*(*v*^3^) to employ all.

The natural question to address is: Which (*v*, 3, 1)-packing should we employ to obtain the bound? To a certain extent, this decision is based on the amount of computational effort that can be expended, and the accuracy of the bound desired. Of course, the best outcome would be to achieve the best accuracy at least computational cost. In certain cases, one might be able to anticipate from exact 2-way alignments when a specific 3-way alignment is likely to lead to an improvement. For example, given three sequences *w*_1_, *w*_2_, *w*_3_, if the 2-way alignments have very different patterns of indels and substitutions, one might expect that the 3-way alignment yields higher cost than the three 2-way alignments. This appears to be difficult to quantify.

In the absence of a good guide to the selection of 3-way alignments that yield the best improvements, two alternatives can be pursued. If accuracy is of paramount concern, we again calculate *all *3-way alignments. Instead of simply averaging their contributions, we assign to each triple a *weight *consisting of the difference between the SP cost for the 3-way alignment and the sum of the three 2-way alignments involved. The best accuracy from the method is obtained by selecting a (*v*, 3, 1)-packing of maximum total weight. This can prove more computationally intensive than simply undertaking all 3-way alignments, since choosing a maximum weight packing is NP-hard [22]. However, there is no need to obtain a packing of maximum weight; one of "large" weight suffices. As a compromise, we develop a simple heuristic (hillclimbing) to choose a packing of large but not necessarily maximum weight in the Methods section.

In the running example, since an (18,3,1)-packing has at most 48 triples, one might hope to obtain a relatively large improvement. The best improvement that we found is 47. Then the hillclimbing algorithm to maximize weight was executed. It yields a collection of 33 triples: four triples with an improvement of 3 each, eleven with an improvement of 2, and thirteen with an improvement of 1. While calculating the average of all 816 alignments increases the lower bound by only 23, this method is comparable in execution time and provides more than twice the improvement in the lower bound.

This demonstrates that, if one is willing to invest the effort to calculate all (v3)
 MathType@MTEF@5@5@+=feaafiart1ev1aaatCvAUfKttLearuWrP9MDH5MBPbIqV92AaeXatLxBI9gBaebbnrfifHhDYfgasaacH8akY=wiFfYdH8Gipec8Eeeu0xXdbba9frFj0=OqFfea0dXdd9vqai=hGuQ8kuc9pgc9s8qqaq=dirpe0xb9q8qiLsFr0=vr0=vr0dc8meaabaqaciaacaGaaeqabaqabeGadaaakeaadaqadaqaauaabeqaceaaaeaacqWG2bGDaeaacqaIZaWmaaaacaGLOaGaayzkaaaaaa@30AB@ 3-way alignments, a simple strategy can be used to select a packing providing a more substantial improvement than that from the elementary lower bound. Nevertheless, it does not save any computation time.

### Heuristic selection of 3-way alignments

If computation time is the more critical, we choose a maximum (*v*, 3, 1)-packing at random, and evaluate the bound. We therefore employ the hillclimbing algorithm (developed in the Methods section) to calculate a packing of maximum size, not using any weight measuring expected improvement at this stage. Then we calculate the optimal 3-way alignment for each triple of the packing, to determine the improvement in the lower bound. Our first attempt gave an SP lower bound of 1968 (an improvement of 22), our second a lower bound of 1966 (an improvement of 20), and our third a lower bound of 1974 (an improvement of 28). There is significant variation in repetitions of the method, as a consequence of the random selections made. Naturally, one cannot conclude much from three trials, so we report the distribution of improvements from 10,000 trials of the method (see Table 2).

**Table 2:** 

Improvement	10	11	12	13	14	15	16	17	18	19	20	21
Frequency	2	6	8	21	64	137	260	422	664	903	1179	1185
Improvement	22	23	24	25	26	27	28	29	30	31	32	33
Frequency	1189	1175	990	724	434	332	172	72	43	14	2	2

It may happen that one is so unlucky as to obtain an improvement of only 10, or lucky enough to obtain an improvement of 33. But one expects, within a very small number of trials, to obtain an improvement of 23 or better. Admittedly, when comparing to an improvement of 47, none of the improvements are striking. To a degree, however, this misses the point. The computation time per trial is much lower than the cost of computing all 3-way alignments. Hence the real benefit is that an improvement can be obtained that is competitive with the average of all 3-way alignments, at a fraction of the cost.

The astute reader can observe that in our 10,000 trials, the average is slightly less than 23, the average of all 816 improvements. This can be attributed to the fact that in a maximum packing on 18 elements, there are nine pairs not appearing in any triple; in a similar experiment with a number of sequences *v *≡ 1, 3 (mod 6) (where the maximum packing has each pair in a triple), the averages would be in closer agreement. Moreover, using a metaheuristic search strategy such as simulated annealing could prove beneficial, again at the expense of a substantial increase in computation time.

## Conclusion

The calculation of *v*-way alignments that are optimal with respect to sum-of-pairs cost is challenging, and substantial effort has been invested in the calculation of upper bounds on SP costs. Exact alignment appears to be computationally out of reach when many long sequences are to be aligned, and lower bounds from sums of exact 2-way alignments are often too weak to be of assistance. In this paper, we establish a generic lower bound using decompositions into fractional alignments. With this we propose two improvements using exact 3-way alignments. The first still calculates all 3-way alignments, but uses a hillclimbing technique to choose a packing among these of maximal weight. This improves on the accuracy of the bound at a similar computational cost to the naive method. The second instead reduces the computation time, by choosing at random a packing, and only then computing the 3-way alignments for the triples of the packing. In our test cases, the improvements are competitive with calculating the overall average, but are achieved at reduced effort in computation.

It remains of interest to determine whether, from exact 2-way alignment data, one can determine which triples are most likely to provide an improvement, prior to calculating the 3-way alignments; see [23] for a related investigation. In our experiment, we did not achieve success better than random by classifying the triples based on features of the 2-way alignments. Nevertheless, such a strategy may augment the effectiveness of the hillclimbing strategy if such a prediction is feasible.

## Methods

Dynamic programming methods for exact 2-way and 3-way alignment that we use are standard [3]. Here we develop the new methods for selecting 3-way alignments used in the computation of lower bounds.

### Hillclimbing 1: Packing Pairs into Triples

The main requirement is to select a random (*v*, 3, 1)-maximum packing. Stinson [24] developed a simple but effective local optimization strategy for the case when the packing T
 MathType@MTEF@5@5@+=feaafiart1ev1aaatCvAUfKttLearuWrP9MDH5MBPbIqV92AaeXatLxBI9gBamrtHrhAL1wy0L2yHvtyaeHbnfgDOvwBHrxAJfwnaebbnrfifHhDYfgasaacH8akY=wiFfYdH8Gipec8Eeeu0xXdbba9frFj0=OqFfea0dXdd9vqai=hGuQ8kuc9pgc9s8qqaq=dirpe0xb9q8qiLsFr0=vr0=vr0dc8meaabaqaciaacaGaaeqabaWaaeGaeaaakeaaimaacqWFtepvaaa@3847@ has exactly *v*(*v *- 1)/6 triples (so that *v *≡ 1, 3 (mod 6)); see also [21,25]. Colbourn and Mathon [26] extended the method to all values of *v*. The basic strategy follows. Start with an empty set T
 MathType@MTEF@5@5@+=feaafiart1ev1aaatCvAUfKttLearuWrP9MDH5MBPbIqV92AaeXatLxBI9gBamrtHrhAL1wy0L2yHvtyaeHbnfgDOvwBHrxAJfwnaebbnrfifHhDYfgasaacH8akY=wiFfYdH8Gipec8Eeeu0xXdbba9frFj0=OqFfea0dXdd9vqai=hGuQ8kuc9pgc9s8qqaq=dirpe0xb9q8qiLsFr0=vr0=vr0dc8meaabaqaciaacaGaaeqabaWaaeGaeaaakeaaimaacqWFtepvaaa@3847@ of triples. Choose two pairs that share a common element, say {*x*, *y*} and {*x*, *z*}, so that neither appears in a triple in T
 MathType@MTEF@5@5@+=feaafiart1ev1aaatCvAUfKttLearuWrP9MDH5MBPbIqV92AaeXatLxBI9gBamrtHrhAL1wy0L2yHvtyaeHbnfgDOvwBHrxAJfwnaebbnrfifHhDYfgasaacH8akY=wiFfYdH8Gipec8Eeeu0xXdbba9frFj0=OqFfea0dXdd9vqai=hGuQ8kuc9pgc9s8qqaq=dirpe0xb9q8qiLsFr0=vr0=vr0dc8meaabaqaciaacaGaaeqabaWaaeGaeaaakeaaimaacqWFtepvaaa@3847@. If the pair {*y*, *z*} also does not appear in a triple of T
 MathType@MTEF@5@5@+=feaafiart1ev1aaatCvAUfKttLearuWrP9MDH5MBPbIqV92AaeXatLxBI9gBamrtHrhAL1wy0L2yHvtyaeHbnfgDOvwBHrxAJfwnaebbnrfifHhDYfgasaacH8akY=wiFfYdH8Gipec8Eeeu0xXdbba9frFj0=OqFfea0dXdd9vqai=hGuQ8kuc9pgc9s8qqaq=dirpe0xb9q8qiLsFr0=vr0=vr0dc8meaabaqaciaacaGaaeqabaWaaeGaeaaakeaaimaacqWFtepvaaa@3847@, we can add the triple {*x*, *y*, *z*} to T
 MathType@MTEF@5@5@+=feaafiart1ev1aaatCvAUfKttLearuWrP9MDH5MBPbIqV92AaeXatLxBI9gBamrtHrhAL1wy0L2yHvtyaeHbnfgDOvwBHrxAJfwnaebbnrfifHhDYfgasaacH8akY=wiFfYdH8Gipec8Eeeu0xXdbba9frFj0=OqFfea0dXdd9vqai=hGuQ8kuc9pgc9s8qqaq=dirpe0xb9q8qiLsFr0=vr0=vr0dc8meaabaqaciaacaGaaeqabaWaaeGaeaaakeaaimaacqWFtepvaaa@3847@ to obtain a packing with one more triple. Suppose instead that {*y*, *z*} does appear in a triple {*w*, *y*, *z*} in T
 MathType@MTEF@5@5@+=feaafiart1ev1aaatCvAUfKttLearuWrP9MDH5MBPbIqV92AaeXatLxBI9gBamrtHrhAL1wy0L2yHvtyaeHbnfgDOvwBHrxAJfwnaebbnrfifHhDYfgasaacH8akY=wiFfYdH8Gipec8Eeeu0xXdbba9frFj0=OqFfea0dXdd9vqai=hGuQ8kuc9pgc9s8qqaq=dirpe0xb9q8qiLsFr0=vr0=vr0dc8meaabaqaciaacaGaaeqabaWaaeGaeaaakeaaimaacqWFtepvaaa@3847@. Removing {*w*, *y*, *z*} from T
 MathType@MTEF@5@5@+=feaafiart1ev1aaatCvAUfKttLearuWrP9MDH5MBPbIqV92AaeXatLxBI9gBamrtHrhAL1wy0L2yHvtyaeHbnfgDOvwBHrxAJfwnaebbnrfifHhDYfgasaacH8akY=wiFfYdH8Gipec8Eeeu0xXdbba9frFj0=OqFfea0dXdd9vqai=hGuQ8kuc9pgc9s8qqaq=dirpe0xb9q8qiLsFr0=vr0=vr0dc8meaabaqaciaacaGaaeqabaWaaeGaeaaakeaaimaacqWFtepvaaa@3847@ and adding {*x*, *y*, *z*} leaves the number of triples in T
 MathType@MTEF@5@5@+=feaafiart1ev1aaatCvAUfKttLearuWrP9MDH5MBPbIqV92AaeXatLxBI9gBamrtHrhAL1wy0L2yHvtyaeHbnfgDOvwBHrxAJfwnaebbnrfifHhDYfgasaacH8akY=wiFfYdH8Gipec8Eeeu0xXdbba9frFj0=OqFfea0dXdd9vqai=hGuQ8kuc9pgc9s8qqaq=dirpe0xb9q8qiLsFr0=vr0=vr0dc8meaabaqaciaacaGaaeqabaWaaeGaeaaakeaaimaacqWFtepvaaa@3847@ unchanged. Nevertheless, the collection of pairs not appearing in a triple of T
 MathType@MTEF@5@5@+=feaafiart1ev1aaatCvAUfKttLearuWrP9MDH5MBPbIqV92AaeXatLxBI9gBamrtHrhAL1wy0L2yHvtyaeHbnfgDOvwBHrxAJfwnaebbnrfifHhDYfgasaacH8akY=wiFfYdH8Gipec8Eeeu0xXdbba9frFj0=OqFfea0dXdd9vqai=hGuQ8kuc9pgc9s8qqaq=dirpe0xb9q8qiLsFr0=vr0=vr0dc8meaabaqaciaacaGaaeqabaWaaeGaeaaakeaaimaacqWFtepvaaa@3847@*does *change, and hence when this process is repeated, choosing a different set of two pairs may permit the addition of a new triple. The method therefore never chooses to reduce the number of triples selected, and relies on "lateral" moves to enable (ultimately) the addition of a new triple when that is possible. Stinson [24] established that this method is very effective in practice, and Gibbons [25] enumerates many problems to which it has been applied.

We describe the method more formally next. We must determine in advance the number of triples in a maximum packing, to provide a stopping criterion. It is well known that in a maximum *(v*, 3, 1)-packing, the number of pairs not appearing in triples is 0 when *v *≡ 1, 3 (mod 6); 4 when *v *≡ 5 (mod 6); v2
 MathType@MTEF@5@5@+=feaafiart1ev1aaatCvAUfKttLearuWrP9MDH5MBPbIqV92AaeXatLxBI9gBaebbnrfifHhDYfgasaacH8akY=wiFfYdH8Gipec8Eeeu0xXdbba9frFj0=OqFfea0dXdd9vqai=hGuQ8kuc9pgc9s8qqaq=dirpe0xb9q8qiLsFr0=vr0=vr0dc8meaabaqaciaacaGaaeqabaqabeGadaaakeaadaWcaaqaaiabdAha2bqaaiabikdaYaaaaaa@2F23@ when *v *≡ 0,2 (mod 6); and v+22
 MathType@MTEF@5@5@+=feaafiart1ev1aaatCvAUfKttLearuWrP9MDH5MBPbIqV92AaeXatLxBI9gBaebbnrfifHhDYfgasaacH8akY=wiFfYdH8Gipec8Eeeu0xXdbba9frFj0=OqFfea0dXdd9vqai=hGuQ8kuc9pgc9s8qqaq=dirpe0xb9q8qiLsFr0=vr0=vr0dc8meaabaqaciaacaGaaeqabaqabeGadaaakeaadaWcaaqaaiabdAha2jabgUcaRiabikdaYaqaaiabikdaYaaaaaa@30F7@ when *v *≡ 4 (mod 6) (see [21]). This underlies the computation of triplesRemaining in the algorithm given next.

   if *v *is odd then

      if *v *≡ 1, 3 (mod 6) then

         *ε *:= 0

      else *ε *: 8;

      *triplesRemaining *: = (*v*(*v *- 1) - *ε*)/6;

   else *triples Remaining *: = *floor*(*v*(*v *- 2)/6);

   *D *: ∅;

   **while ***triplesRemaining *> 0 **do**

   **begin**

      Pick a random element *x *appearing < (*v *- 2)/2 times in *D*;

      Pick random pairs {*x*, *y*}, {*x*, *z*} not appearing in triples in *D*;

      **if **pair {*y*, *z*} appears in a triple *T *of *D*

      **then begin**

            *D *: = *D *-{*T*};

            *triplesRemaining *: = *triplesRemaining *+ 1

         **end**;

   *D *: *D *∪ {{*x*, *y*, *z*}};

   *triplesRemaining *: = *triplesRemaining *- 1

end

Intuitively, the method repeatedly chooses two pairs with a common element, {*x*, *y*} and {*x*, *z*}, neither appearing in a chosen triple. If {*x*, *z*} appears in no triple as well, we can simply add the triple {*x*, *y*, *z*}. Otherwise we delete the unique triple containing {*y*, *z*} before making the addition. The method is a *hillclimbing *technique because it never reduces the number of triples chosen.

The while loop in this method employs as its only stopping criterion that it finds a maximum packing. Stinson [24] incorporates a count of the number of times a "lateral" move is made since the addition of the last triple. When this exceeds a predetermined threshold, the computation is abandoned and restarted. We incorporate this in our implementation as well.

Repeatedly employing this method yields different *(v*, 3, 1)-packings. As each is generated, the relevant 3-way alignments can be calculated to determine the lower bound obtained from this packing. There is a natural tradeoff between computation time and the quality of the bound. Only after a packing is chosen does one need to compute the 3-way alignments involved, giving a factor of *O*(*v*) savings in computation time; however, what has been sacrificed, potentially, is the amount of improvement since the method does not consider (or even know) the improvement until the packing is fully chosen. Of course, one can apply the method repeatedly increasing the computational expense, but with the expectation of improving the accuracy.

### Hillclimbing 2: Maximizing weight

Stinson's method can also be adapted to choose a packing whose improvement is "large". To do this, we require that all 3-way alignments have been calculated. Then we modify the hillclimbing method in two ways. First, we only adjoin a new triple if it makes a nonzero improvement (for this purpose, we can look up the precomputed 3-way alignment cost). Secondly, we only replace one triple by another if it makes an improvement at least as large as the one being removed. One might also consider replacing two or three triples by one, if this yields an improvement. We did not obtain any overall benefit by allowing this. This variant of the Stinson method essentially enables us to find packings of large weight rather than with many triples; it does, however, require that all weights (improvements) be precomputed.

## Authors' contributions

Both authors contributed in all phases of the research.
